# Infant and neonatal deaths in Poland in 1995-2015

**DOI:** 10.34763/devperiodmed.20172102.104110

**Published:** 2017-08-11

**Authors:** Katarzyna Szamotulska, Ewa Mierzejewska

**Affiliations:** 1 Zakład Epidemiologii i Biostatyki, Instytut Matki i Dziecka, Warszawa, Polska

**Keywords:** umieralność niemowląt, umieralność okołoporodowa, czas trwania ciąży, Polska, epidemiologia, martwe urodzenia, Infant mortality, perinatal mortality, questational age, Poland, epidemiology, stillbirths

## Abstract

**Aim:**

The aim of the study was the analysis of trends of infant and neonatal mortality in Poland in 1995-2015, overall and by gestational age, main groups of causes and age at death.

**Material and methods:**

Data from birth and death certificates from 1995, 2000, 2005, 2010, 2014 and 2015 were used. Infant, neonatal, postneonatal, perinatal and gestational age – specific mortality rates were presented. Main groups of causes of deaths were determined according to the International Classification of Diseases ICD-10.

**Results:**

In Poland, in 1995-2015 infant mortality decreased more than three-fold, similarly to neonatal and postneonatal mortality. Early neonatal mortality decreased almost four-fold, stillbirths rate - twofold and perinatal mortality - almost three-fold. The progress, to the greatest extend was related to preterm births. Infant mortality in this group decreased from 128.5 per 1000 preterm live births in 1995 to 36.8 in 2015. The main causes of all infant deaths are perinatal conditions and congenital anomalies.

**Conclusions:**

The decrease of infant and neonatal mortality has been continued in the last twenty years and affected mainly preterm births born from the shorter and shorter gestations, what implicates growing demand for long lasting health care and rehabilitation. Deaths of infants and neonates born at term and not related to congenital anomalies are relatively rare and need individual assessment.

## Streszczenie

**Cel:**

Celem opracowania jest analiza dynamiki umieralności niemowląt i noworodków w Polsce w okresie dwudziestolecia 1995-2015, z uwzględnieniem czasu trwania ciąży, głównych grup przyczyn oraz wieku w momencie zgonu.

**Materiał i metody:**

W pracy wykorzystano zgłoszenia urodzenia noworodka i karty zgonów niemowląt z lat 1995, 2000, 2005, 2010, 2014 i 2015. Obliczono współczynniki umieralności niemowląt, współczynniki umieralności noworodków, współczynniki umieralności ponoworodkowej, współczynniki umieralności okołoporodowej, cząstkowe współczynniki umieralności według czasu trwania ciąży oraz współczynniki i strukturę umieralności w podziale na główne grupy przyczyn według ICD-10.

**Wyniki:**

W okresie ostatnich 20 lat umieralność niemowląt w Polsce zmniejszyła się ponad trzykrotnie, podobnie jak umieralność noworodków i umieralność w okresie ponoworodkowym. Prawie czterokrotnie zmniejszyła się także umieralność w pierwszym tygodniu życia i dwukrotnie – współczynnik martwych urodzeń oraz prawie trzykrotnie – umieralność okołoporodowa. Dokonany postęp dotyczył przede wszystkim noworodków urodzonych przedwcześnie, w której to grupie umieralność niemowląt zmniejszyła się ze 128,5 na 1000 urodzeń żywych w 1995 roku do 36,8 w 2015 roku. Głównymi przyczynami zgonów niemowląt i noworodków są choroby okresu okołoporodowego i wady wrodzone.

**Wnioski:**

W ostatnim dwudziestoleciu, postęp w zakresie zmniejszania umieralności niemowląt był kontynuowany. Wyrażał się on przede wszystkim zwiększaniem szans przeżycia noworodków urodzonych z ciąż o coraz krótszym czasie trwania. Oznacza to, że zwiększa się populacja dzieci wymagających nierzadko długotrwałej opieki medycznej i rehabilitacji. Zgony niemowląt i noworodków urodzonych o czasie i z przyczyn innych niż wady wrodzone nie występują często. Konieczne jest monitorowanie indywidualnych przypadków takich zgonów.

## Wstęp

Umieralność niemowląt jest prawdopodobnie jednym z najdawniej używanych (negatywnych) wskaźników stanu zdrowia – obrazującym stopień, w jakim społeczeństwa są w stanie uchronić dziecko od ryzyka utraty życia. Jest to jednocześnie wskaźnik od bardzo dawna i powszechnie dostępny analizom, z których na przykład wiadomo, że na przełomie XIX i XX wieku na ziemiach polskich umierało co 5 niemowlę, w latach 20-tych XX wieku współczynnik umieralności niemowląt wynosił około 150 na 1000 urodzeń żywych, w 1950 roku – 111, w 1960 roku – 55, w 1990 roku – 19 a dziś – 4. Podobne do zróżnicowania w czasie, widoczne jest zróżnicowanie umieralności niemowląt między krajami. Przeciętnie, w najbiedniejszych krajach świata współczynnik umieralności niemowląt kształtuje się obecnie na poziomie około 50 zgonów na 1000 urodzeń żywych, podczas gdy w najbogatszych – około 5 [1].

W krajach rozwiniętych, w efekcie przemian cywilizacyjnych, w tym technologicznych jakie zachodziły w ostatnich dziesięcioleciach, ryzyko zgonu dziecka w pierwszym roku życia jest niewielkie a zgon dziecka urodzonego o czasie staje się powoli zdarzeniem rzadko występującym. Dlatego może, od pewnego czasu uznano, że w krajach rozwiniętych kwestie zdrowia dziecka i matki zostały rozwiązane i wobec wciąż nie w pełni opanowanych problemów zdrowotnych dorosłych (np. nowotwory, choroby układu krążenia), nie zasługują na zwiększoną uwagę.

Współczynniki umieralności niemowląt, współczynniki umieralności noworodków czy współczynniki umieralności okołoporodowej nadal odzwierciedlają jednak zmiany, jakie zachodzą w sferze opieki zdrowotnej a ich zróżnicowanie sugeruje kierunki działań, które powinny być podjęte w celu zapobiegania dramatycznym zdarzeniom prowadzącym do śmierci dziecka.

Oczywiście, jest pożądane, aby wskaźniki stanu zdrowia najmłodszej populacji opierać nie tylko na zgonach, ale także na zachorowaniach a nawet rozwijać wskaźniki pozytywne, ale zanim to osiągniemy, musimy dokonywać jak najgłębszych analiz danych, które są dostępne.

Niniejsza publikacja nawiązuje do wcześniejszych ogólnokrajowych analiz umieralności niemowląt i noworodków, zdrowia matek oraz opieki okołoporodowej prowadzonych przez Instytut Matki i Dziecka od ponad 50 lat a następnie artykułów pierwszej autorki na ten temat i prac badawczych wykonywanych przez nią w ramach projektu Unii Europejskiej Peristat [2, 3, 4]. Opracowanie otwiera cykl poświęcony zagadnieniom szeroko rozumianej epidemiologii perinatalnej. Przedstawiono tu, między innymi dynamikę cząstkowych współczynników umieralności według czasu trwania ciąży. Dotychczas, w naszych publikacjach współczynniki cząstkowe prezentowane były według grup masy urodzeniowej. Wydaje się jednak, że jakość danych dotyczących czasu trwania ciąży w zgłoszeniach urodzenia noworodka (tzw. najlepsze oszacowanie położnika) jest obecnie na tyle wysoka, że czas trwania ciąży może być uwzględniany w analizach umieralności niemowląt i noworodków. To bowiem czas trwania ciąży a nie masa urodzeniowa *per se* świadczy przede wszystkim o dojrzałości noworodka i determinuje jego szanse przeżycia poza organizmem matki.

W niniejszym opracowaniu przyczyny zgonów niemowląt i noworodków przedstawiono tylko według głównych rozdziałów Międzynarodowej Statystycznej Klasyfikacji Chorób i Problemów Zdrowotnych. Stosowanie tej klasyfikacji do bezpośredniej analizy szczegółowych pojedynczych przyczyn zgonów noworodków i niemowląt od lat nie było uznawane za uzasadnione, ponieważ niektóre pojedyncze przyczyny znajdujące się w różnych rozdziałach klasyfikacji, faktycznie reprezentują wspólną etiologię, która nie jest widoczna, gdy przyczyny te analizowane są w rozproszeniu. Na przykład, niektóre choroby uwarunkowane genetycznie są kodowane nie tylko w rozdziale dotyczącym wad wrodzonych, ale i w rozdziale dotyczącym chorób układu nerwowego oraz w rozdziale dotyczącym zaburzeń wydzielania wewnętrznego, stanu odżywienia i przemian metabolicznych. W ostatnich latach powstały nowe propozycje grupowania pojedynczych przyczyn zgonów noworodków [5, 6] i w przyszłej publikacji poświęconej szczegółowym przyczynom zgonów w 2014 roku, wykorzystana zostanie jedna z tych propozycji – ujęcie hierarchiczne.

## Cel pracy

Celem opracowania jest analiza dynamiki umieralności niemowląt i noworodków w Polsce w okresie dwudziestolecia 1995-2015, z uwzględnieniem czasu trwania ciąży, z której urodził się noworodek, głównych grup przyczyn oraz wieku w momencie zgonu.

## Materiał i metody

W pracy wykorzystano zgłoszenia urodzenia noworodka i karty zgonów niemowląt z lat 1995, 2000, 2005, 2010, 2014 i 2015. Dokumenty te, po wypełnieniu przez pracowników służby zdrowia i pracowników Urzędów Stanu Cywilnego, są przekazywane do Głównego Urzędu Statystycznego, gdzie są przetwarzane głównie dla celów statystyki ludności. Instytut Matki i Dziecka od ponad 30 lat wykorzystuje zanonimizowane indywidualne dane ze zgłoszeń urodzenia noworodka jako narzędzie służące monitorowaniu stanu zdrowia niemowląt, noworodków i matek na poziomie ogólnokrajowym. W Polsce istnieje jeszcze jedno źródło danych dotyczące zgonów okołoporodowych – sprawozdawczość w formie zagregowanej ze szpitali (formularz MZ-29), jednak liczba zgonów w wieku 0-6 dni w tym źródle jest niedoszacowana, ponieważ sprawozdawczość nie uwzględnia ewentualnych zgonów po przekazaniu noworodka do innego szpitala.

Obliczono współczynniki umieralności niemowląt (liczba zgonów w pierwszym roku życia na 1000 urodzeń żywych), współczynniki umieralności noworodków (liczba zgonów w wieku 0-27 dni na 1000 urodzeń żywych, współczynniki umieralności ponoworodkowej (liczba zgonów niemowląt w wieku ponad 27 dni na 1000 urodzeń żywych), współczynniki umieralności okołoporodowej (suma liczby zgonów w wieku 0-6 dni i martwych urodzeń na 1000 urodzeń żywych i martwych), cząstkowe współczynniki umieralności według czasu trwania ciąży (liczba zgonów w danej grupie czasu trwania ciąży w stosunku do liczby urodzeń żywych w tej grupie czasu trwania ciąży) oraz współczynniki i strukturę umieralności w podziale na główne grupy przyczyn według Międzynarodowej Statystycznej klasyfikacji Chorób i Problemów Zdrowotnych (rewizja dziesiąta, ICD-10). Wyodrębniono grupę „Wybranych stanów rozpoczynających się w okresie okołoporodowym” (P00-P96) oraz grupę „Wad rozwojowych wrodzonych, zniekształceń i aberracji chromosomowych” (Q00-Q99). Przyczyn zgonów nie uwzględniono dla roku 1995, ponieważ wówczas stosowana była inna – dziewiąta rewizja klasyfikacji.

Zgodnie z przyjętą na świecie a także w Polsce praktyką współczynniki obliczono dzieląc liczbę zgonów zarejestrowanych w danym roku kalendarzowym przez liczbę urodzeń żywych zarejestrowanych w tym samym roku kalendarzowym.

Braki danych dotyczących czasu trwania ciąży dla urodzeń żywych i zgonów uzupełniono proporcjonalnie według znanego rozkładu czasu trwania ciąży. Dla urodzeń żywych uzupełnienia te dotyczyły 3005 przypadków w 1995 roku, 95 –w 2000 roku, 33 –w 2005 roku, 2 –w 2010 roku, 5 –w 2014 roku i 1589 –w 2015 roku. Dla zgonów uzupełnienia te dotyczyły 298 przypadków w 1995 roku, 59 –w 2000 roku, 37 –w 2005 roku, 2 – w 2010 roku, 1 –w 2014 roku i 8 –w 2015 roku.

Wyniki podano zarówno dla 2014 roku, jak i 2015 roku, ponieważ w 2015 roku, na skutek wprowadzenia błędnego rozporządzenia w sprawie wzorów karty urodzenia i karty martwego urodzenia (Dz.U. 2015, poz.171) przestały być dostępne dane dotyczące martwych urodzeń. Podany tu nieporównywalny współczynnik umieralności okołoporodowej i martwych urodzeń w 2015 roku pochodzi ze wspomnianej wcześniej rutynowej zagregowanej sprawozdawczości Ministerstwa Zdrowia [7].

## Wyniki

W okresie ostatnich 20 lat umieralność niemowląt w Polsce zmniejszyła się ponad trzykrotnie (z 13,6 zgonów na 1000 urodzeń żywych w 1995 roku do 4,0 w 2015 roku), podobnie jak umieralność noworodków (z 10,1 zgonów na 1000 urodzeń żywych w 1995 roku do 2,9 w 2015 roku) i umieralność w okresie ponoworodkowym (z 3,5 na 1000 urodzeń żywych w 1995 roku do 1,1 w 2015 roku (tab, I). Prawie czterokrotnie zmniejszyło się także ryzyko zgonu w pierwszym tygodniu życia (z 8,0 na 1000 urodzeń żywych w 1995 roku do 2,1 w 2015 roku) oraz dwukrotnie – współczynnik martwych urodzeń (z 7,3 na 1000 urodzeń w 1995 roku do 3,6 w 2014 roku). Te dwie ostatnie składowe: umieralność w pierwszym tygodniu życia i współczynnik martwych urodzeń tworzą łącznie umieralność okołoporodową, która także uległa prawie trzykrotnemu obniżeniu (z 15,3 na 1000 urodzeń w 1995 roku do 5,6 w 2014 roku).

**Tabela I:** Umieralność niemowląt, noworodków i okołoporodowa w latach 1995-2015. Table I. Infant, neonatal and perinatal mortality in 1995-2015.

	Urodzenia *Births*				Zgony niemowląt/*Infant deaths*										Zgony okołoporodowe	
Rok *Year*	ogółem *total*	żywe *live*	martwe *still*		ogółem *total*		0-27 dni *0-27 days*		0-6 dni *0-6 days*		7-27 dni *7-27 days*		>27 dni *>27 days*		*Perinatal deaths*	
	n	n	n	wsp.^1^	n	wsp.^2^	n	wsp.^2^	n	wsp.^2^	n	wsp.^2^	n	wsp.^2^	n	wsp.^1^
	*n*	*n*	*n*	*rate^1^*	*n*	*rate^2^*	*n*	*rate^2^*	*n*	*rate^2^*	*n*	*rate^2^*	*n*	*rate^2^*	*n*	*rate^1^*
1995	437001	433795	3206	7,3	5891	13,6	4361	10,1	3486	8,0	875	2,0	1530	3,5	6692	15,3
2000	380476	378348	2128	5,6	3067	8,1	2116	5,6	1563	4,1	553	1,5	951	2,5	3691	9,7
2005	366095	364383	1712	4,7	2340	6,4	1633	4,5	1233	3,4	400	1,1	707	1,9	2945	8,0
2010	415030	413300	1730	4,2	2057	5,0	1454	3,5	1087	2,6	367	0,9	603	1,5	2817	6,8
2014	376501	375160	1341	3,6	1583	4,2	1084	2,9	781	2,1	303	0,8	499	1,3	2122	5,6
2015*	*370383*	369308	*1075*	*2,9*	1476	4,0	1067	2,9	762	2,1	305	0,8	409	1,1	*1837*	*5,0*

n – liczba bezwzględna, wsp.^1^ – współczynnik na 1000 urodzeń ogółem, wsp.^2^ – współczynnik na 1000 urodzeń żywych, * – szacunkowe dane o martwych urodzeniach n – absolute number, rate^1^ – rate per 1000 total births, rate^2^ – rate per 1000 live births, * – estimated data for stillbirths

Główną determinantą ryzyka zgonu w pierwszym roku życia i w pierwszym miesiącu życia jest czas trwania ciąży, z której urodził się noworodek. Ryzyko zgonu niemowlęcia urodzonego z porodu przedwczesnego (≤36 tygodnia ciąży) jest ponad dwudziestokrotnie wyższe, niż w przypadku dziecka urodzonego o czasie (≥37 tygodnia ciąży). Im krótszy jest czas trwania ciąży, tym mniejsze są szanse przeżycia. Jeszcze 20 lat temu (w 1995 roku) w okresie niemowlęcym umierało około 80% dzieci urodzonych przed 28 tygodniem ciąży, 30% dzieci urodzonych w 28-31 tygodniu, około 8% dzieci urodzonych w 32-34 tygodniu i około 3% dzieci urodzonych w 35,36 tygodniu (tab. II). Dziś (2015 rok) szanse przeżycia takich noworodków radykalnie zwiększyły się i zagrożenie zgonem w okresie niemowlęcym wynosi odpowiednio 44, 7, 2 i 1%.

**Tabela II:** Umieralność niemowląt według czasu trwania ciąży w latach 1995-2015. Table II. Gestational age – specific infant mortality in 1995-2015.

	Czas trwania ciąży *Gestational age*														
	22-27 tyg. *22-27 weeks*			28-31 tyg. *28-31 weeks*			32-34 tyg. *32-34 weeks*			35-36 tyg. *35-36 weeks*			37 tyg. i powyżej *37 weeks and above*		
Rok *Year*	urodzenia żywe *live births*	zgony niemowląt *infant deaths*		urodzenia żywe *live births*	zgony niemowląt *infant deaths*		urodzenia żywe *live births*	zgony niemowląt *infant deaths*		urodzenia żywe *live births*	zgony niemowląt *infant death*s		urodzenia żywe *live births*	zgony niemowląt *infant deaths*	
	n	n	wsp.	n	n	wsp.	n	n	wsp.	n	n	wsp.	n	n	wsp.
	*n*	*n*	*rate*	*n*	*n*	*rate*	*n*	*n*	*rate*	*n*	*n*	*rate*	*n*	*n*	*rate*
1995	1818	1397	795,2	3586	1062	307,4	7284	591	84,4	16085	479	31,2	401973	2028	5,3
2000	1268	739	592,3	2715	478	178,9	5564	317	58,0	14310	283	20,1	354393	1189	3,4
2005	1230	675	555,3	2437	328	136,6	5591	263	47,7	14407	209	14,7	340675	818	2,5
2010	1371	680	496,7	2637	261	99,0	6394	196	30,7	16677	160	9,6	386216	754	2,0
2014	1282	559	436,0	2675	180	67,3	6764	154	22,8	16483	140	8,5	347940	539	1,6
2015	1226	545	445,2	2401	168	70,1	6722	140	20,9	16260	114	7,0	341095	488	1,4

n – liczba bezwzględna, wsp.– współczynnik na 1000 urodzeń żywych o danym czasie trwania ciąży n – absolute number, rate^1^ – rate per 1000 live births at given gestational age

U dzieci urodzonych o czasie zgon w okresie niemowlęcym był i pozostaje zdarzeniem rzadko występującym, chociaż w tej grupie stanowiącej ogół wszystkich rodzonych żywo dzieci (około 93%), notuje się także postęp wyrażony spadkiem umieralności niemowląt z 5,3 na 1000 urodzeń żywych ≥37 tygodnia ciąży w 1995 roku do 1,4 w 2015 roku.

W związku z radykalnym zmniejszaniem się ryzyka zgonu w grupie dzieci urodzonych przedwcześnie, co dotyczy zwłaszcza okresu noworodkowego, można zastanawiać się nad tym, czy zmniejszenie ryzyka zgonu bezpośrednio po urodzeniu nie skutkuje zwiększeniem ryzyka zgonu w dalszym okresie życia niemowlęcia.

Wydaje się, że tak nie jest, ponieważ nie obserwuje się jednoczesnego zwiększenia umieralności w okresie ponoworodkowym tych niedojrzałych i nierzadko chorych dzieci (ryc. 1). Uwzględnienie w obliczeniach umieralności ponoworodkowej tylko dzieci, które przeżyły okres noworodkowy nie zmienia wnioskowania (dane nie przedstawione).

**Ryc. 1:**
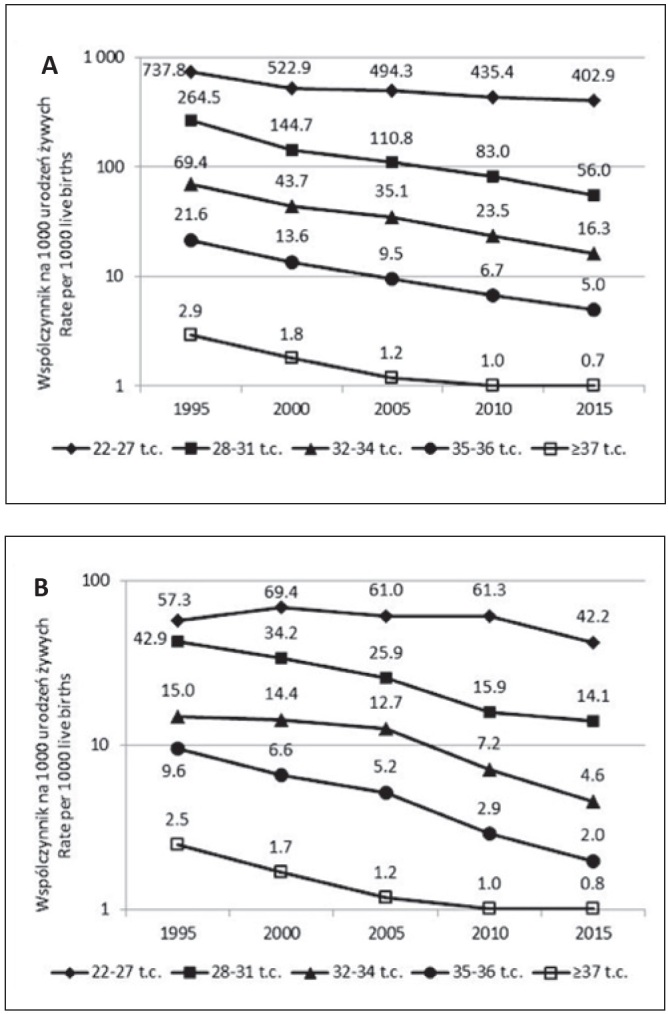
Umieralność niemowląt według czasu trwania ciąży (t.c.) i wieku w momencie zgonu w latach 1995-2015. (A) 0-27 dni, (B) powyżej 27 dni. Fig. 1. Infant mortality by gestational age (g.a.) and age at deaths in 1995-2015. (A) 0-27 days (B) above 27 days.

Głównymi przyczynami zgonów noworodków i niemowląt są choroby okresu okołoporodowego, zwłaszcza związane z wcześniactwem (około 50% zgonów niemowląt i 60% zgonów noworodków) oraz wady wrodzone (około 30% zgonów niemowląt i noworodków) (tab. III).

**Table III:** Infant mortality by main groups of causes in 2000-2015. Tabela III. Umieralność niemowląt według głównych grup przyczyn w latach 2000-2015.

Rok	Ogółem *Total*			Wady wrodzone *Congenital malformations*			Choroby okresu okołoporodowego *Perinatal conditions*			Pozostałe *Other*		
*Year*	n	wsp.	%	n	wsp.	%	n	wsp.	%	n	wsp.	%
	n	rate	%	n	rate	%	n	rate	%	n	rate	%
2000	3067	8,1	100.0%	1042	2,8	34,0%	1460	3,9	47,6%	565	1,5	18,4%
2005	2340	6,4	100.0%	800	2,2	34,2%	1170	3,2	50,0%	370	1,0	15,8%
2010	2057	5,0	100.0%	699	1,7	34,0%	1059	2,6	51,5%	299	0,7	14,5%
2014	1583	4,2	100.0%	588	1,6	37,1%	803	2,1	50,7%	192	0,5	12,1%
2015	1476	4,0	100.0%	515	1,4	34,9%	780	2,1	52,8%	181	0,5	12,3%

n – liczba bezwzględna, wsp.– współczynnik na 1000 urodzeń żywych n – absolute number, rate– rate per 1000 live births

W okresie ponoworodkowym zgony z powodu wad wrodzonych stanowią około 45%, zgony związane z następstwami stanów powstałych w okresie okołoporodowym – 20% i zgony ze wszystkich pozostałych przyczyn - 35%. Obserwując dynamikę zgonów niemowląt, można zauważyć, że spadek ryzyka zgonu następuje niezależnie od grupy wyjściowej przyczyny.

Odmiennie jednak kształtują się przyczyny zgonów dzieci urodzonych przedwcześnie i dzieci urodzonych o czasie (szczegółowe dane nie są przedstawione w tabelach). Noworodki urodzone przedwcześnie, o ile umierają, to najczęściej w następstwie wcześniactwa (ponad 70% zgonów w 2015 roku, z czego ponad 85% z powodu zaburzeń związanych ze skróconym czasem trwania ciąży i opóźnionym rozwojem płodu – kody P05, P07 klasyfikacji ICD-10). To właśnie w zakresie obniżenia się umieralności z powodu chorób okresu okołoporodowego u niemowląt przedwcześnie urodzonych – z 51,4 zgonów na 1000 urodzeń żywych przed 37 tygodniem ciąży w 2000 roku do 26,8 w 2015 roku – dokonano w ostatnim 20-leciu największy postęp.

Ponadto, ponad 20% zgonów noworodków urodzonych przedwcześnie powodowanych jest wadami rozwojowymi wrodzonymi, zniekształceniami i aberracjami chromosomowymi (2015 rok), z czego prawie 30% wrodzonymi wadami rozwojowymi układu krążenia a zwłaszcza serca (kody Q20-Q28 klasyfikacji ICD-10).

Zgon noworodka urodzonego o czasie – o ile następuje, to głównie z powodu wady wrodzonej (ponad 50% przyczyn zgonów w tej grupie w 2015 roku, z czego 50% stanowią wrodzone wady rozwojowe układu krążenia a zwłaszcza serca), ale także z powodu wszystkich pozostałych przyczyn, w tym okołoporodowych, na przykład niedotlenienia wewnątrzmacicznego i zamartwicy urodzeniowej (ponad 30% tzw. przyczyn okołoporodowych w grupie noworodków urodzonych o czasie w 2015 roku) czy zespołu nagłej śmierci niemowląt (ponad 20% pozostałych przyczyn zgonów w tej w grupie noworodków). Ogólnie, zgony niemowląt urodzonych o czasie zdarzają się rzadko a przyczyny tych zgonów są bardzo zróżnicowane (ryc. 2).

**Ryc. 2:**
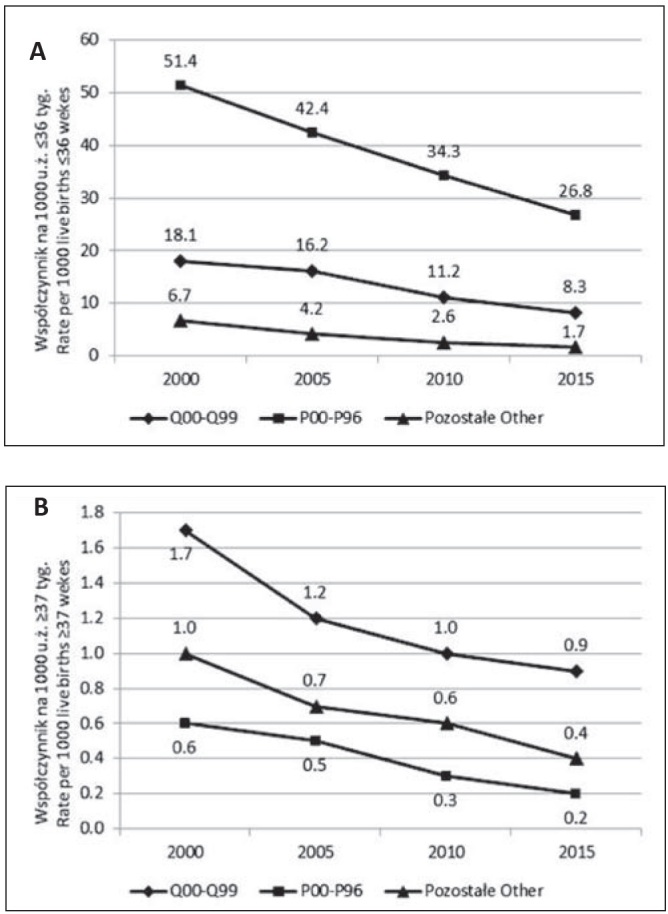
Umieralność niemowląt według czasu trwania ciąży i głównych grup przyczyn w latach 2000-2015. (A) – niemowlęta urodzone przed 37 tygodniem ciąży, (B) – niemowlęta urodzone w 37 tygodniu trwania ciąży i później. Q00-Q99: Wady rozwojowe wrodzone, zniekształcenia i aberracje chromosomowe;P00-P96: Wybrane stany rozpoczynające się w okresie okołoporodowym. Fig. 2. Infant mortality by gestational age and main groups of causes in 2000-2015. (A) – infants born before 37 weeks gestational age (B) – infants born in 37 weeks gestational age and later. Q00-Q99: Congenital malformations, deformations and chromosomal abnormalities;P00-P96: Certain conditions originating in the perinatal period.

## Dyskusja

Umieralność niemowląt w Polsce w ciągu ostatnich 20 lat uległa znacznemu obniżeniu i trendy spadkowe dotyczyły zarówno umieralności noworodkowej jak i w okresie ponoworodkowym. Największy spadek obserwowany był w latach 1995-2005: umieralności noworodkowej o 4,5 na 1000 urodzeń żywych a umieralności postneonatalnej o 1,0 na 1000 urodzeń żywych. W latach następnych obniżanie obu umieralności miało wolniejsze tempo, bardziej zbliżone do liniowego: odpowiednio 0,9 i 0,5 w okresie 5 lat.

Polska zbliża się stopniowo do poziomu osiąganego przez kraje Europy Zachodniej (kraje Unii Europejskiej przed 2004 rokiem). W roku 1995 współczynniki umieralności neonatalnej wynosiły 10,1 na 1000 urodzeń żywych dla Polski i 3,7 dla krajów Europy Zachodniej, natomiast w roku 2000 - odpowiednio 5,6 oraz 3,1 na 1000 urodzeń żywych [8]. Podobnie współczynnik umieralności postneonatalnej w 1995 roku wynosił dla Polski 3,5 a dla krajów Europy Zachodniej – 2,0 na 1000 urodzeń, natomiast w roku 2000 – odpowiednio 2,5 i 1,6 na 1000 urodzeń żywych. Jednak z Raportu EURO-PERISTAT wynika, że chociaż Polska w roku 2010 w porównaniu z rokiem 2004 dokonała postępu w zakresie obniżenia współczynników umieralności noworodkowej i postneonatalnej, to nadal była jednym z krajów o najwyższej umieralności noworodków (3,5 na 1000 urodzeń żywych) i niemowląt (5,0 na 1000 urodzeń żywych), a kraje z wyższymi niż Polska współczynnikami to Łotwa, Malta, Rumunia i Północna Irlandia (jako część Wielkiej Brytanii) oraz Węgry (tylko w zakresie umieralności niemowląt) [9]. Liderami w tym zestawieniu są kraje skandynawskie, ale także np. Słowenia czy Estonia, ze współczynnikami umieralności noworodkowej poniżej 2 i umieralności niemowląt poniżej 3 na 1000 urodzeń żywych.

W Polsce na przestrzeni ostatnich 20 lat obserwowane jest obniżenie umieralności zarówno wśród niemowląt donoszonych, jak i przedwcześnie urodzonych. Największe zmiany – ponad 4-krotne zmniejszenie ryzyka zgonu – dotyczyły niemowląt urodzonych w 28-31, 32-34 i 35-36 tygodniu ciąży. Prawie 4-krotne zmniejszenie – dość niskiego już – ryzyka zgonu miało też miejsce w grupie niemowląt urodzonych po 36 tygodniu ciąży. Stosunkowo najmniej, choć również znacząco bo prawie 2-krotnie obniżyło się ryzyko zgonu niemowląt urodzonych najwcześniej – pomiędzy 22 i 27 tygodniem ciąży. Jednakże MacDorman i wsp., który porównał umieralność niemowląt z 2010 roku według czasu trwania ciąży w wybranych krajach europejskich i Stanach Zjednoczonych stwierdził, że współczynniki w Polsce są 2-3-krotnie wyższe niż w innych krajach rozwiniętych [10]. Można przyjąć, że Polska pod tym względem jest opóźniona w stosunku do krajów Europy Zachodniej o około 10 lat.

Jednak z drugiej strony należy także zauważyć, że ponieważ obniżenie umieralności dzieci urodzonych przedwcześnie nie jest odzwierciedleniem zmniejszenia częstości występowania wcześniactwa w Polsce (odsetek urodzeń żywych z porodów przed 37 tygodniem trwania ciąży jest dość stabilny w ciągu ostatnich 20 lat i wynosił 6-7% w tym okresie), to skutkuje większą przeżywalnością tej grupy dzieci, często wymagających długotrwałej opieki i rehabilitacji. Fakt ten niesie za sobą implikacje dla organizacji opieki zdrowotnej i systemu +nansowania ochrony zdrowia w kraju.

Główne przyczyny umieralności niemowląt w Polsce to choroby okresu okołoporodowego i wady wrodzone. Odpowiadają one odpowiednio za ok. 50% i 30% zgonów w trakcie pierwszego roku życia i proporcje te nie zmieniały się znacząco w trakcie ostatnich 15 lat.

Około 30% wynosi także odsetek zgonów noworodkowych z powodu wad wrodzonych w Polsce prezentowany w raporcie EURO-PERISTAT z roku 2010, co stawia ją na 9 miejscu spośród 27 krajów (regionów) pod względem udziału wad jako przyczyny zgonów w tym okresie [9]. Jednak należy mieć na względzie, że w części krajów niski udział wad wrodzonych wśród przyczyn umieralności niemowląt wynika z częstszego indukowania poronień w następstwie wykrycia wady wrodzonej u płodu.

Dane z Rejestru Wielkopolskiego – pełnoprawnego członka sieci rejestrów wad wrodzonych EUROCAT pokazują, że Polska należy do krajów o przeciętnej częstości występowania wad wrodzonych (24,9 na 1000 urodzeń – lata 2006-2010) [11]. Powodem może być dość młody wiek kobiet rodzących w Polsce (11,4% kobiet powyżej 35 roku życia) w porównaniu z innymi krajami europejskimi (mediana europejska wynosi 19,4% matek powyżej 35 roku życia). Dodatkowo w ostatnich kilku latach obserwowana jest tendencja spadkowa częstości występowania wad wrodzonych w rejestrze Wielkopolskim (z 30,0 w roku 2000 do 18,0 na 1000 urodzeń w roku 2012). Dlatego też współistniejące trendy spadkowe w występowaniu i umieralności niemowląt z powodu wad wrodzonych nie pozwalają na oszacowanie, czy w Polsce zwiększa się przeżywalność dzieci z wadami wrodzonymi.

Analiza zmian umieralności noworodków i niemowląt w Polsce w czasie oraz porównania międzynarodowe pokazują z jednej strony sukcesy – zdecydowaną poprawę wskaźników umieralności dotyczącą wszystkich analizowanych subpopulacji w tej grupie wieku w ciągu ostatnich 20 lat. Z drugiej jednak strony odstęp, jaki wciąż dzieli Polskę od krajów Europy Zachodniej wymaga działań w zakresie dwóch głównych przyczyn odpowiedzialnych za zdrowie niemowląt: chorób okresu okołoporodowego i wad wrodzonych. Konieczna jest dalsza poprawa systemu opieki zdrowotnej w zakresie wczesnej diagnostyki i skutecznej terapii, także w okresie ciąży. Jednocześnie, występuje pilna potrzeba zintensyfikowania prac badawczych nad możliwościami zapobiegania wcześniactwu i wadom wrodzonym. Istotna jest też pogłębiona analiza zgonów dzieci wypisywanych po urodzeniu ze szpitala jako zdrowe, zwłaszcza w aspekcie potencjalnych uwarunkowań społeczno-ekonomicznych.

## Wnioski

1. W ostatnim dwudziestoleciu, postęp w zakresie zmniejszania umieralności niemowląt jest kontynuowany. Wyraża się on przede wszystkim zwiększaniem szans przeżycia dzieci urodzonych z ciąż o coraz krótszym czasie trwania.
2. Głównymi przyczynami zgonów niemowląt i noworodków jest wcześniactwo i wady wrodzone. Natężenie zgonów z powodu wcześniactwa maleje, przy braku zmian w zakresie jego występowania. Oznacza to, że zwiększa się populacja dzieci wymagających nierzadko długotrwałej opieki medycznej i rehabilitacji. Jednocześnie, występuje pilna potrzeba zintensyfikowania prac badawczych nad możliwościami zapobiegania wcześniactwu i wadom wrodzonym.
3. Zgony niemowląt i noworodków urodzonych o czasie i z przyczyn organicznych innych niż wady wrodzone nie występują często. Konieczne jest monitorowanie indywidualnych przypadków takich zgonów.
